# mGluR5 regulates REST/NRSF signaling through N-cadherin/β-catenin complex in Huntington’s disease

**DOI:** 10.1186/s13041-020-00657-7

**Published:** 2020-08-28

**Authors:** Jéssica M. de Souza, Khaled S. Abd-Elrahman, Fabiola M. Ribeiro, Stephen S. G. Ferguson

**Affiliations:** 1 University of Ottawa Brain and Mind Institute and Department of Cellular and Molecular Medicine, University of Ottawa, 451 Smyth Road, Ottawa, Ontario K1H 8M5 Canada; 2 Department of Biochemistry and Immunology, ICB, Universidade Federal de Minas Gerais, Belo Horizonte, Brazil; 3 Department of Pharmacology and Toxicology, Faculty of Pharmacy, Alexandria University, Alexandria, 21521 Egypt

**Keywords:** Huntington’s disease, REST/NRSF, Wnt pathway, mGluR5, Src, zQ175, BACHD

## Abstract

Repressor element 1-silencing transcription factor/neuron-restrictive silencer factor (REST/NRSF) is a transcription repressor and its expression is regulated by the Wnt pathway through β-catenin. Metabotropic glutamate receptor 5 (mGluR5) signaling plays a key role in controlling neuronal gene expression. Interestingly, REST/NRSF nuclear translocation and signaling, as well as mGluR5 signaling are altered in the presence of mutant huntingtin. It remains unclear whether mGluR5 can modulate Wnt and REST/NRSF signaling under physiological conditions and whether this modulation is altered in Huntington’s disease (HD). Using primary corticostriatal neurons derived from wild type mouse embryos, we find that targeting mGluR5 using the agonist, DHPG, or the negative allosteric modulator, CTEP, modulates REST/NRSF expression by regulating the assembly of N-cadherin/ β-catenin complex in a Src kinase-dependent manner. We have validated our in vitro findings in vivo using two HD mouse models. Specifically, we show that pharmacological inhibition of mGluR5 in *zQ*175 mice and genetic ablation of mGluR5 in BACHD mice corrected the pathological activation of Src and rescued REST/NRSF-dependent signaling. Together, our data provide evidence that mGluR5 regulates REST/NRSF expression via the Wnt pathway and highlight the contribution of impaired REST/ NRSF signaling to HD pathology.

## Introduction

Huntington’s disease (HD) is a hereditary autosomal dominant neurodegenerative disease caused by an unstable expansion of over 35 glutamines in the amino-terminus of the huntingtin (HTT) protein [1, 2]. Although both wild-type and mutated HTT (mHTT) proteins are ubiquitously expressed, HD is associated with selective neuronal loss. Degeneration occurs preferentially in the striatum and cortex and, in later stages, it extends to a variety of brain regions, including the hippocampus and hypothalamus [3, 4]. As a result, HD symptoms include cognitive alterations, psychiatric abnormalities and motor dysfunction [4, 5]. To date, there are no disease-modifying treatments for HD, mostly due to the lack of full understanding of the physiological functions of HTT and the pathological cascade initiated by mHTT in the brain. However, HTT is found to be crucial for neuronal survival, vesicle transport, calcium homeostasis, and transcriptional regulation [6].

Previous studies have identified the repressor element 1-silencing transcription factor/neuron-restrictive silencer factor (REST/NRSF) as a key regulator of HTT-mediated gene expression [7–9]. REST/NRSF target genes are mainly involved in neuronal development and synaptic transmission and their expression is found to be dysregulated in HD [8]. Specifically, HTT sequesters REST/NRSF in the cytoplasm and prevents it from forming the nuclear co-repressor complex at the neuron-restrictive silencer element (RE1/NRSE) nuclear site, thereby facilitate gene transcription. On the other hand, mHTT is incapable of retaining REST/NRSF in the cytosol leading to the pathological entry of REST/NRSF into the nucleus and inhibition of gene transcription [7–9]. REST/NRSF expression is regulated by the canonical Wnt/β-catenin pathway [10–12]. β-catenin is usually retained at the plasma membrane in a complex with the cytoplasmic portion of cadherins [13–15]. Phosphorylation of β-catenin disrupts its interaction with cadherins leading to either β-catenin cytosolic degradation or its translocation to the nucleus where it can regulate REST/NRSF transcription [16, 17].

The metabotropic glutamate receptor 5 (mGluR5) is a G_αq_-coupled receptor and it has been shown to regulate cadherin/β-catenin complex assembly in the vascular endothelium [17]. However, the molecular link(s) between mGluR5 and cadherin/β-catenin complex formation and whether mGluR5 can regulate REST/NRSF expression and signaling in neurons remains unclear. Additionally, we and others have shown that mGluR5 plays a key role in the progression of HD in mouse models of the disease [18–20]. However, it remains to be defined whether mGluR5-mediated regulation of N-cadherin/ β-catenin signaling and REST-dependent gene expression is altered in HD.

In the present study, we show that mGluR5 regulates N-cadherin phosphorylation via Src kinase to alter N-cadherin/β-catenin assembly and REST/NRSF expression in mouse primary corticostriatal neurons. Moreover, we show that in 15 month old zQ175 mice, enhanced Src and N-cadherin phosphorylation can be corrected by chronic pharmacological inhibition of mGluR5 using the negative allosteric modulator (NAM), CTEP. This is paralleled by a reduction in REST/NRSF and consequent increase in its target gene synaptosomal nerve-associated protein 25 (SNAP-25) expression in CTEP-treated zQ175 mice. These findings are also validated in a BACHD mouse model of HD as we show that genetic ablation of mGluR5 reduces REST/NRSF and increases SNAP25 mRNA and protein expression. Thus, our results show that mGluR5 regulates REST/NRSF expression and signaling and highlight the relevance of Wnt pathway in HD progression and therapeutics.

## Results

### mGluR5 modulates N-cadherin phosphorylation in primary neuronal cultures

**Fig. 1:**
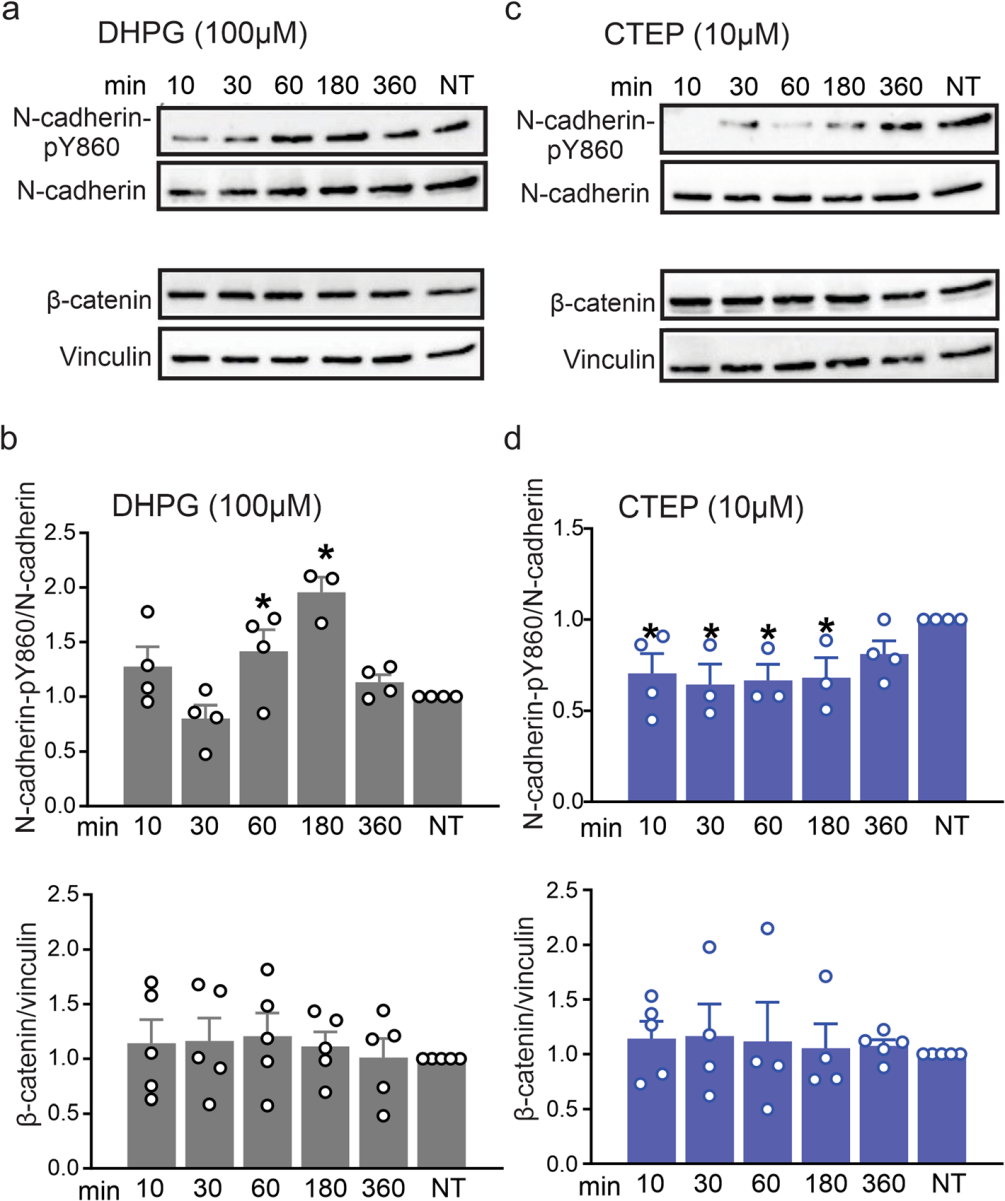
mGluR5 modulates N-cadherin phosphorylation but not β-catenin expression in primary neuronal cultures. Representative western blots and quantification of fold change of N-cadherin phosphorylation at Y860 (pY860) and β-catenin expression with the corresponding loading controls in primary cultured corticostriatal neurons derived from E15 wild-type mouse embryos stimulated with either the mGluR group I agonist DHPG (100 μM) **(a** and **b)** or the mGluR5-selective NAM, CTEP (10 μM) **(c** and **d)** for 10, 30, 60, 180 and 360 mins. N-cadherin-pY860 was normalized to total N-cadherin, and β-catenin was normalized to vinculin (*n* = 4–5 for each group). Values represent mean ± SEM and are expressed as a fraction of the non-treated (NT) cultures. * denotes *P* < 0.05 versus NT cultures. Statistical significance was assessed by one-way ANOVA and Fisher’s LSD multiple comparisons

Several previous studies demonstrated the importance of mGluR5 in the pathophysiology of HD [18–21]. Moreover, mGluR5 was shown to modulate cadherin/β-catenin association in the vascular endothelium [17]. Since the involvement of Wnt pathway in the development of HD is not well-known, we tested whether the N-cadherin/ β-catenin complex was modulated by mGluR5 in neuronal cells and whether this modulation was affected by mHTT. To start with, in vitro experiments were performed using primary corticostriatal neuronal cultures derived from wild-type E15 mouse embryos. Neuronal cultures were treated with either 100 μM (S)-3,5-Dihydroxyphenylglycine (DHPG), a group I mGluR agonist, or 10 μM 2-chloro-4-((2,5-dimethyl-1-(4-(trifluoromethoxy)phenyl)-1H-imidazol-4-yl)ethynyl) pyridine (CTEP), a mGluR5-specific NAM, for 10, 30, 60, 180 and 360 min. DHPG induced significant N-cadherin phosphorylation at Y860 following treatment for 60 and 180 min (Fig. 1a and b), whereas CTEP reduced N-cadherin-pY680 phosphorylation following 10, 30, 60 and 180 min of treatment compared to non-treated neurons (Fig. 1c and d). Total levels of β-catenin protein expression were not changed by either DHPG (Fig. 1a and b) or CTEP (Fig. 1c and d) treatment. These results indicated that mGluR5 could trigger N-cadherin phosphorylation without affecting β-catenin expression. Therefore, it was likely that mGluR5 activation modulated the assembly of N-cadherin/β-catenin complex, as this association was known to be tightly-regulated by phosphorylation of N-cadherin [16, 17].

### mGluR5 modulates N-cadherin/β-catenin complex through Src kinase in primary neuronal cultures

**Fig. 2:**
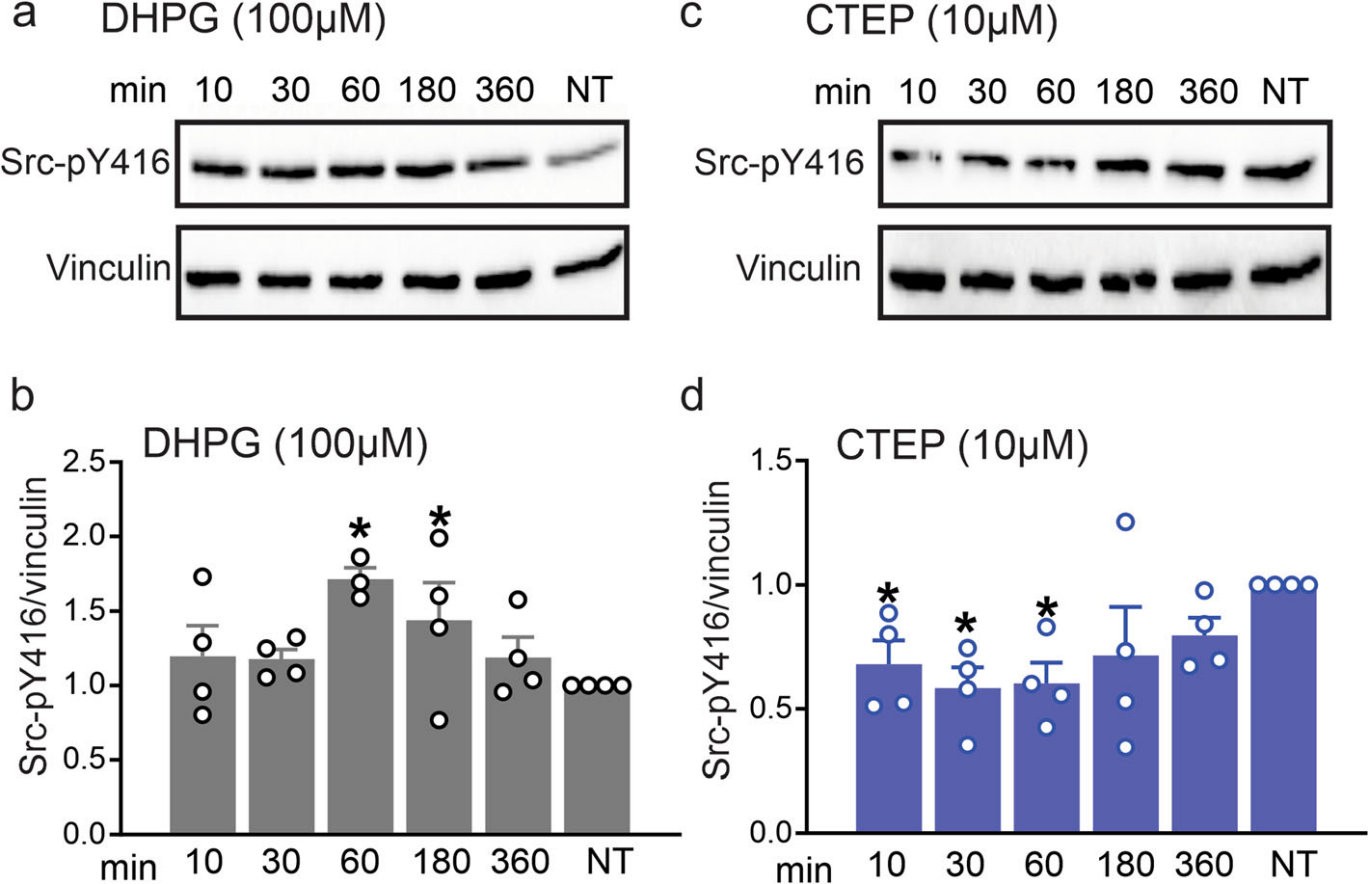
mGluR5 modulates Src phosphorylation expression in primary neuronal cultures. Representative western blots and quantification of fold change Src phosphorylation at Y416 (pY416) with the corresponding loading controls in primary cultured corticostriatal neurons from E15 wild-type mouse embryos stimulated with either the mGluR group I agonist DHPG (100 μM) (**a** and **b)** or mGluR5-selective NAM, CTEP (10 μM) (**c** and **d)** for 10, 30, 60, 180 and 360 mins. pSrc was normalized to vinculin (*n* = 4 for each group). Values represent mean ± SEM and are expressed as a fraction of the non-treated (NT) cultures. * *P* < 0.05 vs NT cultures. Statistical significance was assessed by one-way ANOVA and Fisher’s LSD multiple comparisons

We next tested the mechanism by which mGluR5 triggered N-cadherin-pY680 phosphorylation to disrupt N-cadherin/β-catenin interaction. Previously published studies highlighted the regulatory role of Src kinase in cadherin/β-catenin dissociation [22–25]. Since Src kinase was also demonstrated to be a downstream substrate activated by mGluR5 [26–28], we analyzed Src phosphorylation at Y416 in neuronal cultures treated with either 100 μM DHPG or 10 μM CTEP for 10, 30, 60, 180 and 360 min. DHPG induced Src phosphorylation following 60 and 180 min of treatment (Fig. 2a and b), whereas CTEP reduced Src phosphorylation after 10, 30 and 60 min of treatment compared to non-treated values (Fig. 2c and d). These results suggested that mGluR5 could activate Src kinase which potentially phosphorylated N-cadherin and regulated the scaffolding of N-cadherin and β-catenin complex.

**Fig. 3:**
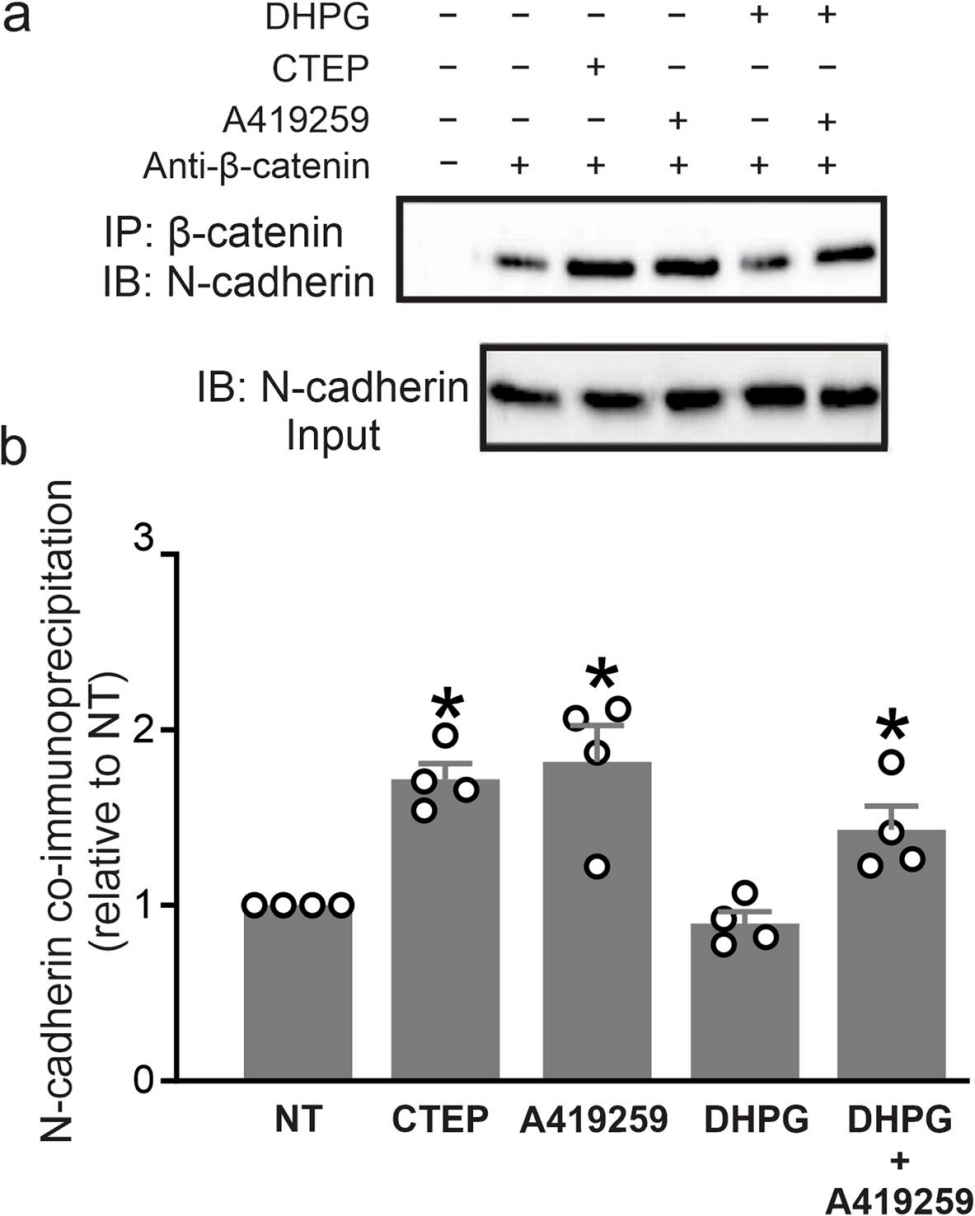
Inhibition of either mGluR5 or Src activity increase the interaction between N-cadherin and β-catenin in primary neuronal cultures. Representative western blots (**a**) and quantification (**b**) for coimmunoprecipitation of N-cadherin with β-catenin **(upper panel)** in primary cultured corticostriatal neurons from E15 wild-type mouse embryos stimulated with either mGluR5-selective NAM, CTEP (10 μM), Src family kinase inhibitor A419259 (1 μM) or the mGluR group I agonist DHPG (100 μM) for 60 min. **Lower panel (a)** shows N-cadherin lysates used to normalize N-cadherin co-immunoprecipitation with β-catenin (*n* = 4 for each group). Values represent mean ± SEM and are expressed as a fraction of the non-treated (NT) cultures incubated with β-catenin antibody only. *P < 0.05 versus NT cultures. Statistical significance was assessed by one-way ANOVA and Fisher’s LSD multiple comparisons

To assess whether mGluR5-dependent Src phosphorylation of N-cadherin influenced the interaction of N-cadherin with β-catenin, we immunoprecipitated N-cadherin and blotted for co-immunoprecipitated β-catenin in wild-type corticostriatal neurons following 60 min exposure to either 100 μM DHPG, 10 μM CTEP or 1 μM A419259 (a Src family kinase inhibitor). We found that treatment with either CTEP or A419259 increased the interaction between N-cadherin and β-catenin in neurons, whereas a 60 min exposure to DHPG did not alter the interaction between N-cadherin and β-catenin (Fig. 3a and b). More so, addition of DHPG to A419259 did not alter the effects of A419259 on N-cadherin and β-catenin interaction. These results suggested that the inhibition of either mGluR5 or Src signaling could increase N-cadherin and β-catenin complex formation.

### REST/NRSF signaling is modulated by mGluR5 in primary neuronal cultures

**Fig. 4:**
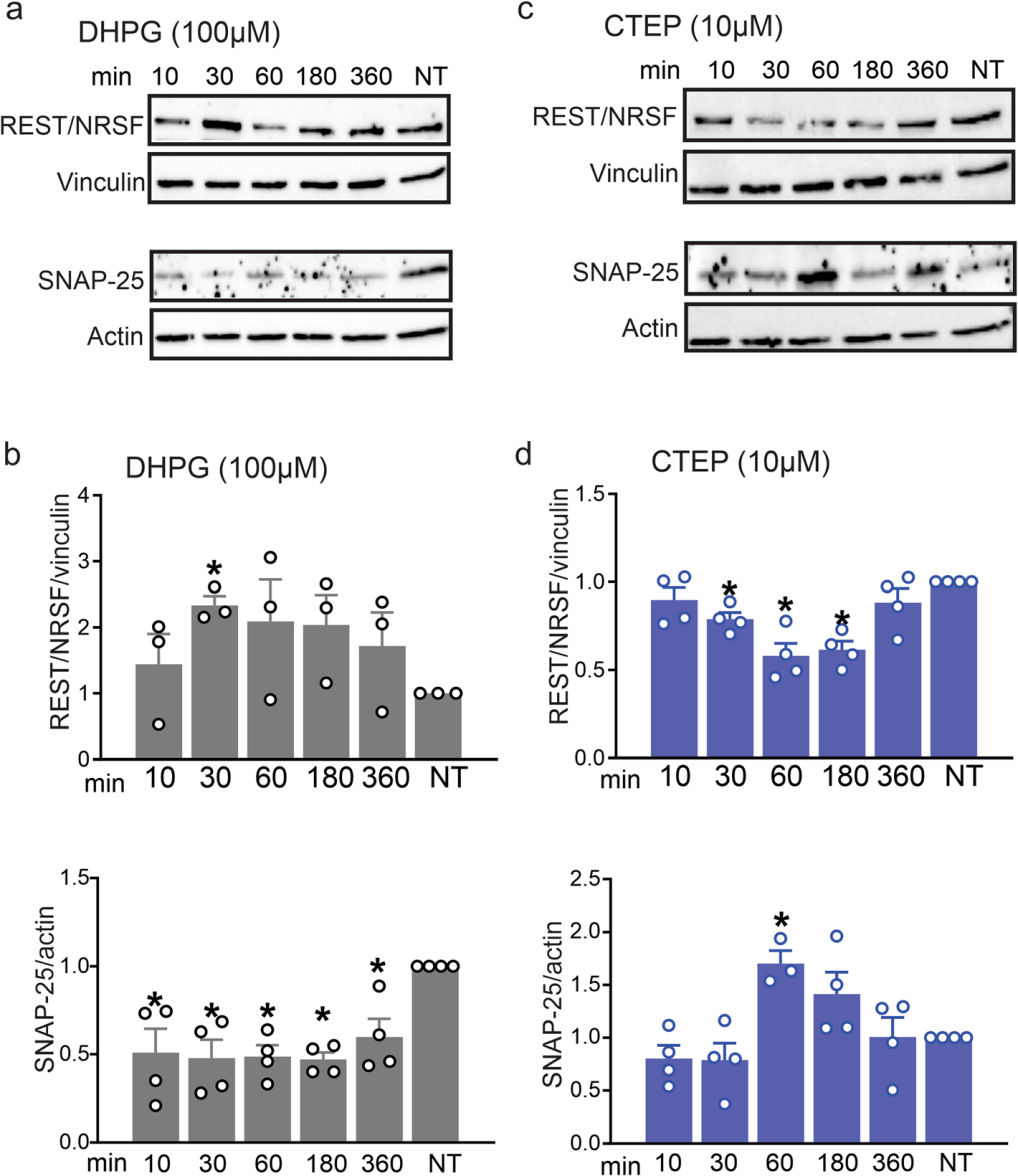
mGluR5 modulates both REST/NRSF and SNAP-25 expression in primary neuronal cultures. Representative western blots and quantification of fold change in REST/NRSF and SNAP-25 protein expression with the corresponding loading controls in primary cultured corticostriatal neurons from E15 wild-type mouse embryos stimulated with either the mGluR group I agonist DHPG (100 μM) (**a** and **b**) or mGluR5-selective NAM, CTEP (10 μM) (**c** and **d**) for 10, 30, 60, 180 and 360 mins. REST/NRSF was normalized to vinculin, and SNAP-25 was normalized to actin (*n* = 3–4 for each group). Values represent mean ± SEM and are expressed as a fraction of the non-treated (NT) cultures. *P < 0.05 versus NT cultures. Statistical significance was assessed by one-way ANOVA and Fisher’s LSD multiple comparisons. REST/NSRF was probed on β-catenin blots and therefore the same vinculin blot is presented for REST/NRSF (Fig. 4a and c) and β-catenin (Fig. 1a and c)

REST/NRSF was previously shown to be an important transcriptional repressor regulated by canonical Wnt pathway and modulates the transcription of genes involved in neurogenesis, neuronal development, and synaptic transmission [12, 29]. Our findings so far suggested the possibility that mGluR5 was signaling via the Wnt/β-catenin pathway. Therefore, we assessed whether either DHPG or CTEP treatment altered the expression of REST/NRSF in neuronal cultures. We found that a 30 min treatment of cultures with DHPG resulted in an increase in REST/NRSF expression (Fig. 4a and b), whereas CTEP treatment for 30, 60 and 180 mins reduced REST/NRSF expression compared to non-treated control cells (Fig. 4c and d). We then tested whether mGluR5-mediated regulation of REST/NRSF expression was paralleled by changes in the expression of SNAP-25, a protein that contains a RE1 transcriptional regulatory sequence and was previously demonstrated to be a key target gene of REST/NRSF [30–32]. We found that DHPG treatment for 10, 30, 60, 180 and 360 min significantly reduced SNAP-25 expression when compared to non-treated cultures (Fig. 4a and b). Conversely, CTEP treatment for 60 min significantly enhanced SNAP-25 expression when compared to non-treated cultures (Fig. 4c and d). Together, these results demonstrated that mGluR5 can regulate REST/NRSF expression and such regulation was paralleled by changes in the expression of its target gene SNAP-25.

### Chronic mGluR5 antagonism alters REST/NRSF signaling via N-cadherin/β-catenin complex in zQ175 mice

**Fig. 5:**
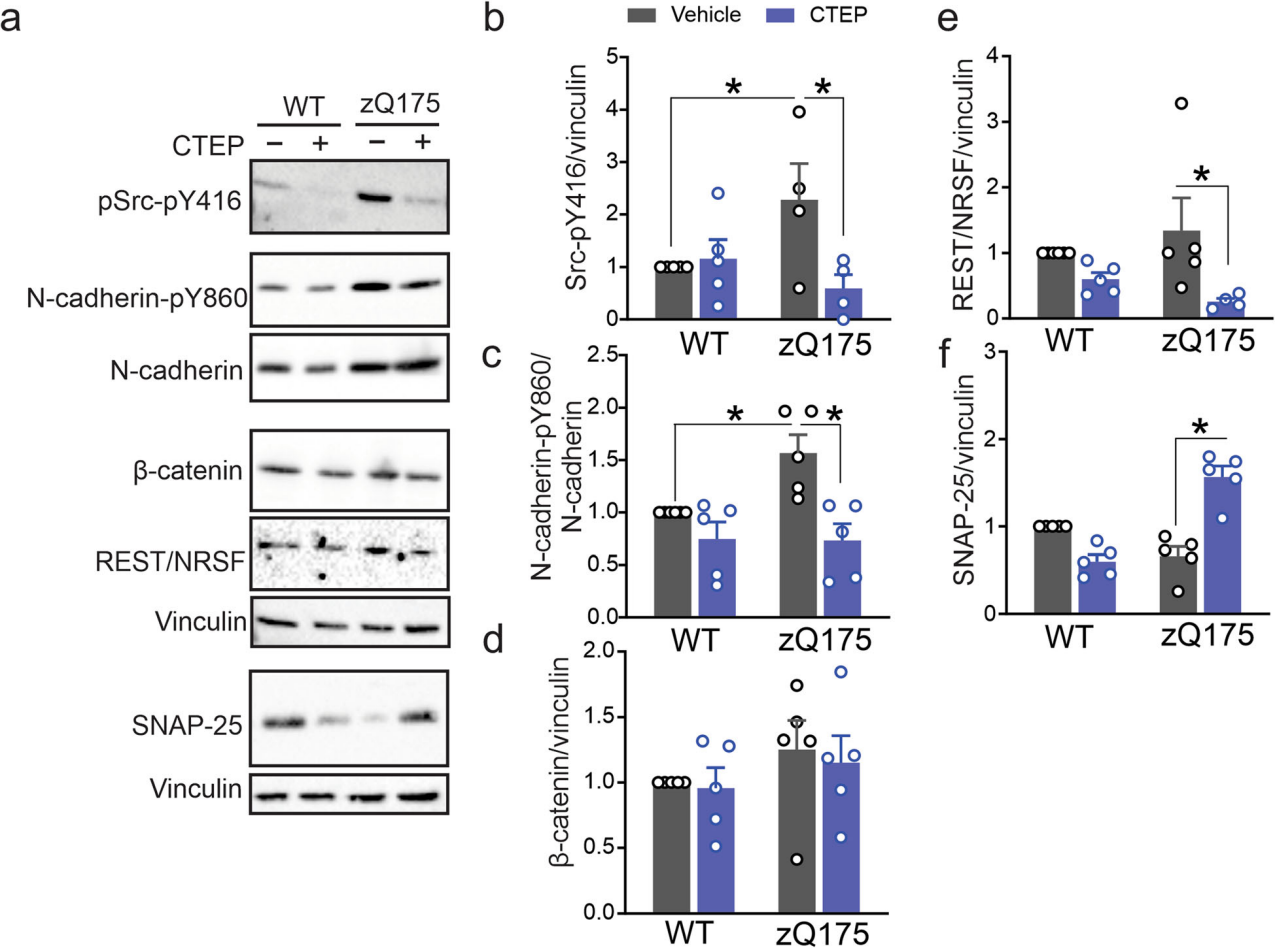
Chronic CTEP treatment modulates REST/NRSF signaling by N-cadherin/β-catenin complex in zQ175 mice. Representative western blots (**a**) and quantification of fold change in Src phosphorylation at Y416 (pY416) (**b**), N-cadherin phosphorylation at Y860 (pY860) (**c**), β-catenin protein expression (**d**), REST/NRSF protein expression (**e**) and SNAP-25 protein expression (**f**) with the corresponding loading controls in brain lysates from heterozygous zQ175 and wild-type (WT) mice after chronic treatment with either vehicle or CTEP (2 mg/kg) for 12 weeks. N-cadherin-pY860 was normalized to total N-cadherin, and Src-pY416, β-catenin, REST/NRSF and SNAP-25 were normalized to vinculin (*n* = 5–6 to each group). Values represent mean ± SEM and are expressed as a fraction of the vehicle-treated WT value. * denotes *P* < 0.05 and statistical significance was assessed by two-way ANOVA and Fisher’s LSD multiple comparisons

We previously provided evidence that aberrant mGluR5 signaling played a key role in HD pathology and that both pharmacological and genetic silencing of mGluR5 could rescue motor deficits and mitigate HD pathology in zQ175 and Q111 mouse models, respectively [18, 19]. Therefore, we assessed whether altered REST/NRSF was one of the potential components contributing to pathological mGluR5 signaling in zQ175 mice and whether CTEP treatment could correct REST/NRSF expression and signaling. To do this, we assessed brain lysates from 15 month old male wild-type and zQ175 mice that were treated with either vehicle or CTEP (2 mg/Kg every 48 h) for 12 weeks. We found that Src and N-cadherin phosphorylation was significantly increased in brain lysates derived from vehicle-treated zQ175 mice and was reduced to values comparable to wild-type mouse lysates following chronic CTEP treatment (Fig. 5a-c). In contrast, β-catenin expression was not altered between either wild-type or zQ175 mice that were treated with either vehicle or CTEP (Fig. 5a and d). However, we found that REST/NRSF expression was reduced in CTEP-treated zQ175 mice compared to vehicle-treated mice (Fig. 5a and e) and was associated with a significant increase in SNAP-25 expression in CTEP treated zQ175 mice (Fig. 5a and f). Thus, our in vivo findings in zQ175 mouse model corroborated our in vitro data and confirmed that mGluR5 modulated REST/NRSF expression and its target genes via the Wnt pathway in HD mice.

### mGluR5 modulates REST/NRSF signaling in BACHD mice

**Fig. 6:**
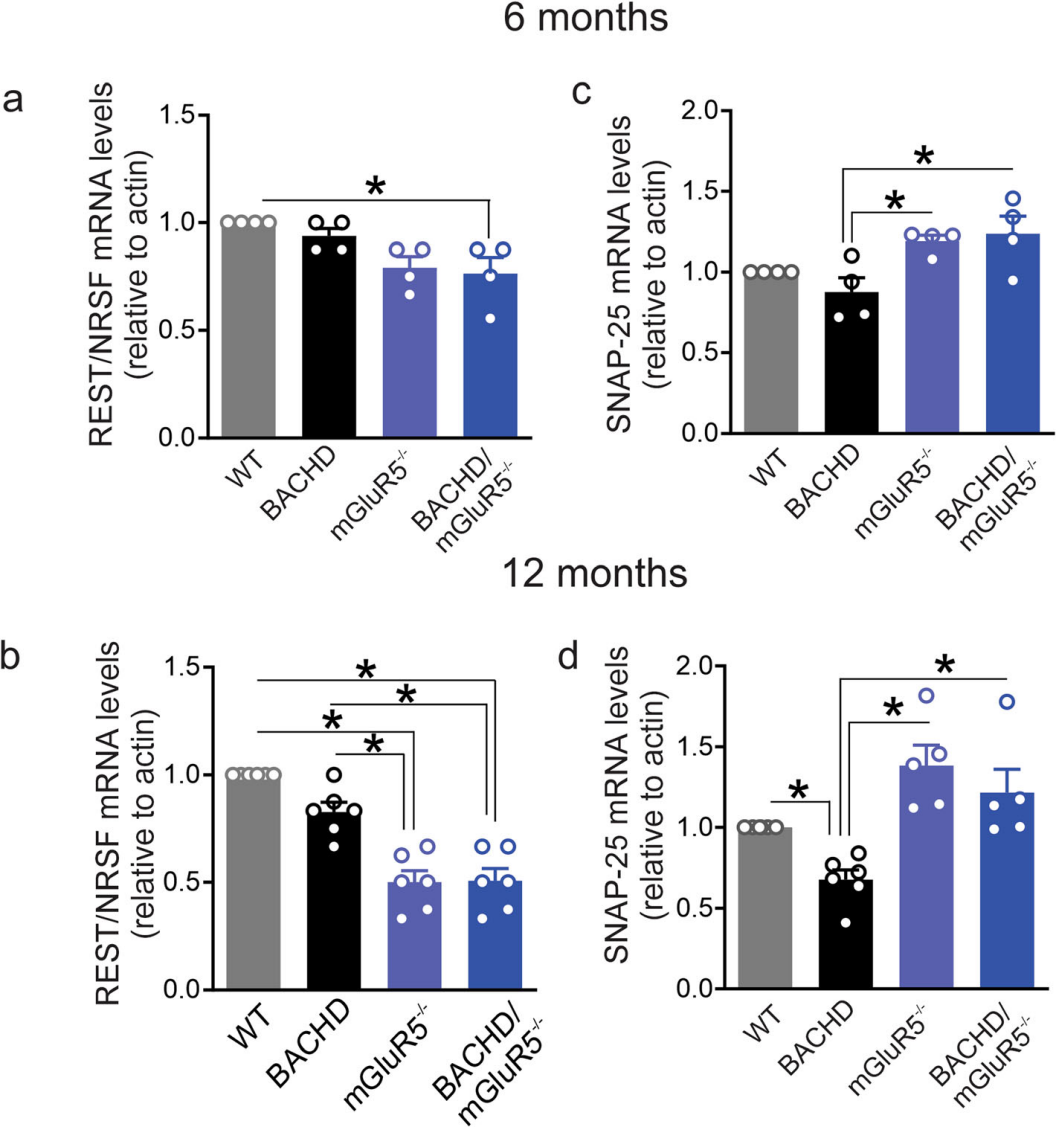
Genetic ablation of mGluR5 modulate REST/NRSF and SNAP-25 mRNA levels in BACHD mice. mRNA levels of REST/NRSF (**a** and **b**) and SNAP-25 (**c** and **d**) in hippocampus samples from wild-type (WT), mGluR5^−/−^, BACHD and BACHD/ mGluR5^−/−^ mice at 6 and 12 months of age. mRNA levels were assessed by quantitative RT-PCR, which was performed in triplicate and normalizes to actin mRNA levels (*n* = 6 to each group). Values represent mean ± SEM and are expressed as a fraction of WT. * denotes *P* < 0.05 and statistical significance was assessed by one-way ANOVA and Fisher’s LSD multiple comparisons

**Fig. 7:**
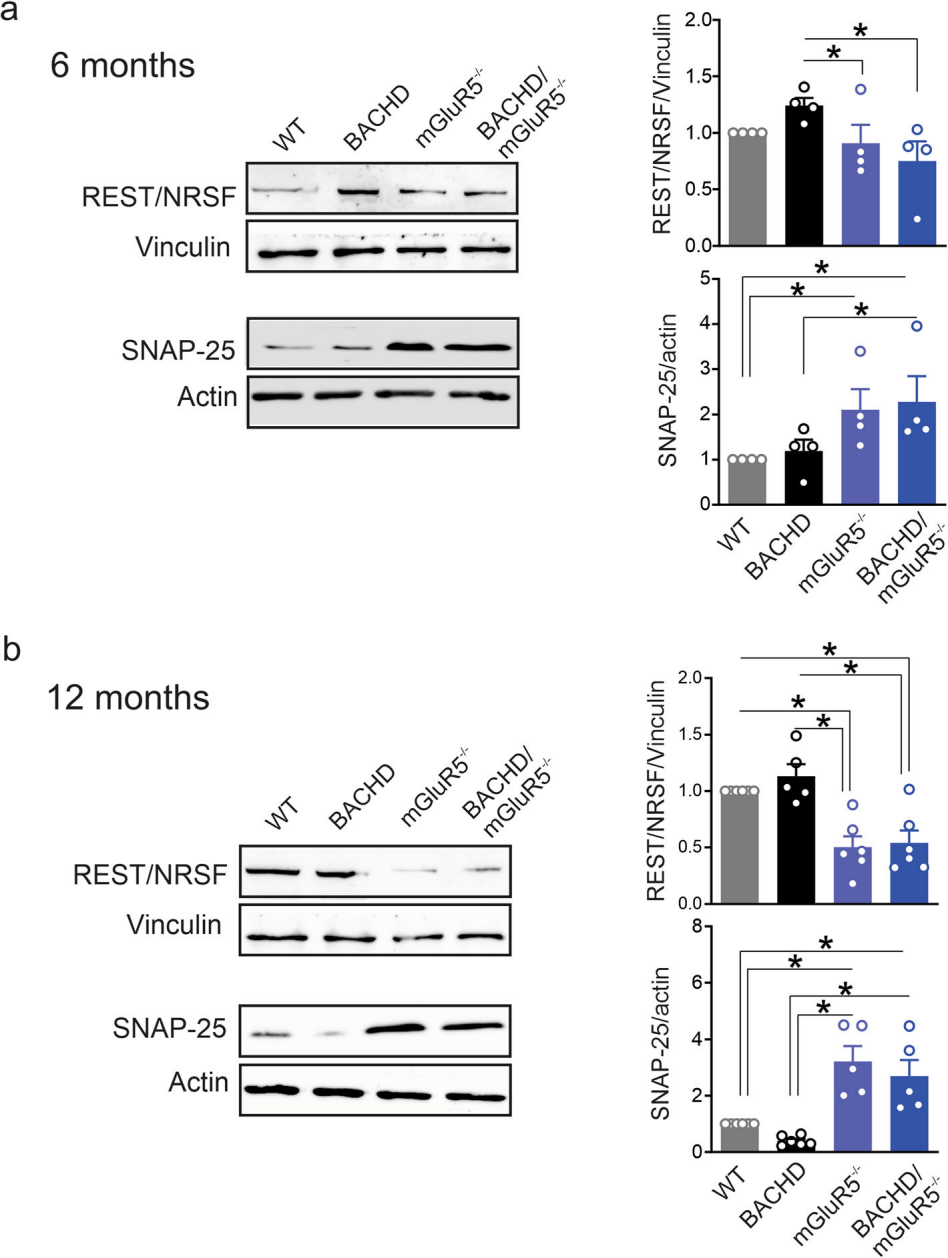
Genetic ablation of mGluR5 modulate REST/NRSF and SNAP-25 protein levels in BACHD mice. Representative western blots and quantification of fold change in protein levels of REST/NRSF and SNAP25 with the corresponding loading controls in hippocampal lysates from wild-type (WT), mGluR5^−/−^, BACHD and BACHD/ mGluR5^−/−^ mice at (**a**) 6 and (**b**) 12 months of age. REST/NRSF was normalized to vinculin and SNAP-25 was normalized to actin (*n* = 4–6 to each group). Values represent mean ± SEM and are expressed as a fraction of WT value. * denotes *P* < 0.05 and statistical significance was assessed by one-way ANOVA and Fisher’s LSD multiple comparisons

To further validate our findings, we assessed REST/NRSF and SNAP-25 mRNA levels in the bacterial artificial chromosome (BAC) HD mouse (BACHD) model, which was previously shown to exhibit a progressive neurodegenerative phenotype [33, 34], in the absence (BACHD/mGluR5^−/−^) and presence of mGluR5 (BACHD). We analyzed hippocampal lysates from both 6 and 12 month old, mice since REST/NRSF was previously demonstrated to be highly expressed in post mitotic hippocampal neurons [35]. We detected a reduction in REST/NRSF mRNA expression in BACHD/mGluR5^−/−^ mice as early as 6 months of age when compared to wild-type mice (Fig. 6a), and found that at 12 months of age both mGluR5^−/−^ and BACHD/mGluR5^−/−^ mice presented with reduced REST/NRSF mRNA expression when compared with both wild-type and BACHD mice (Fig. 6b). SNAP-25 mRNA expression was increased in mGluR5^−/−^ and BACHD/mGluR5^−/−^ mice at 6 and 12 months of age when compared with age-matched BACHD mice, whereas SNAP-25 mRNA was significantly reduced in 12 month old BACHD mice when compared to age-matched wild-type mice (Fig. 6c and d). We finally validated that the changes in mRNA expression were translated at protein level by immunoblotting for REST/NRSF and SNAP-25 proteins in the same group of mice. At 6 months of age, we detected a reduction in REST/NRSF protein in mGluR5^−/−^ and BACHD/mGluR5^−/−^ compared to BACHD mice and an increase in SNAP-25 protein in mGluR5^−/−^ and BACHD/mGluR5^−/−^ compared to wild-type mice (Fig. 7a). REST/NRSF protein levels of 12 month old mGluR5^−/−^ and BACHD/mGluR5^−/−^ mice were significantly reduced, whereas, SNAP-25 levels were significantly increased when compared to age-matched wild-type and BACHD mice (Fig. 7b). Taken together, we were able to validate in a different mouse model and using a genetic silencing approach that mGluR5 is a critical regulator of REST/NSRF signaling in HD.

## Discussion

Emerging evidence indicates that the dysregulation of the transcriptional repressor REST/NRSF cell signaling and the consequent epigenetic remodeling represents a critical mechanism in the progression of the neurodegeneration associated with ischemia and AD [30, 31, 36, 37]. Specifically, an increase in REST/NRSF signaling is linked to neuronal death during ischemia [36, 37]. On the other hand, the loss of REST/NRSF expression is associated with cognitive impairment in AD [30]. Studies in HD have shown that mHTT, contrary to HTT, cannot maintain the cytoplasmic localization of REST/NRSF leading to its nuclear translocation resulting in the repression of many targets genes, including brain-derived neurotrophic factor (BDNF) [7–9, 38–40]. Here, we show that mGluR5 controls REST/NRSF-mediated gene expression by inducing Src-dependent disassembly of the N-cadherin/β-catenin scaffold. We also show that pathological activation of mGluR5 in two distinct mouse models of HD is associated with aberrant REST/NRSF signaling that is mitigated by either genetic or pharmacological silencing of mGluR5. Thus, it is evident that impaired REST/NRSF signaling represents one of the mechanisms by which mGluR5 contributes to HD pathophysiology at the nuclear level.

We provide in vivo and in vitro evidence that Wnt signaling downstream of mGluR5 regulates REST/NRSF expression and its target gene SNAP-25. We also show in two HD mouse models, zQ175 and BACHD, that the inhibition of mGluR5 signaling either pharmacologically or genetically can reduce REST expression and consequently enhance SNAP-25 expression. This is in line with previous work that shows that mHTT causes the pathological entry of REST/NRSF into the nucleus, leading to a transcriptional repression of its target genes such as SNAP-25 [7–9] and that both pharmacological and genetic ablation of mGluR5 reduces mHTT burden in HD mice [18, 19].

The pharmacological and genetic inhibition of mGluR5 is also associated with improved motor function and disease pathology in zQ175 and HdhQ111/Q111 mouse models [18, 19, 41]. Therefore, we propose that defects in mGluR5-regulated REST/NRSF signaling contribute to the pathophysiology of HD. REST/NRSF has been implicated in the regulation of more than 2000 genes within the mammalian genome [42], but only a subset of target genes responsive to REST/NRSF are associated with the widespread neuronal dysfunction in HD [7, 31]. SNAP25 is a t-soluble NSF attachment receptor (SNARE) presynaptic protein, which is involved in the regulation of synaptic vesicle exocytosis [43]. Reduction of SNAP25 expression in brain samples from patients with a higher HD pathological grade has been reported, which is correlated with a defect in the neurotransmitter release machinery [44]. A defect in the pre-synaptic release machinery may also potentially affect other processes of relevance to the pathogenesis of HD, such as BDNF release from cortical neurons that is important for the survival of the striatal medium-sized spiny neurons [45]. Interestingly, BDNF is targeted by REST/NRSF and reduction in BDNF expression has been reported in both mouse models and patients of HD [41, 46, 47]. A decreased synthesis and transport of BDNF is believed to underlie the neuronal loss in the caudate nucleus and the putamen in the dorsal striatum, and the striatum vulnerability could explain the involuntary motor dysfunction characteristic of HD [45, 46, 48]. In line with these reports, we have previously reported that brain BDNF levels are reduced in zQ175 mice in a mGluR5-dependent manner [41]. The modulation of REST/NRSF, its target gene SNAP-25 and BDNF expression by mGluR5 NAM may therefore represent a novel pharmacological tool to halt the progression of HD and potentially other neurodegenerative diseases.

Targeting mGluR5 did not alter in vitro expression of β-catenin, the key factor in Wnt pathway that induces REST/NRSF expression, and we did not detect any change in β-catenin expression in either vehicle or CTEP-treated zQ175 mice. Activation of tyrosine kinases is known to drive β-catenin translocation to the nucleus and promote its binding to the TCF/LEF family of transcription factors to facilitate gene expression. Specifically, Src kinase can regulate N-cadherin/β-catenin association and, consequently, the nuclear accumulation of β-catenin [49, 50]. Previous work in melanoma cells showed that Src activation leads to N-cadherin phosphorylation at Y860 resulting in the subsequent uncoupling of β-catenin [50]. Interestingly, Src kinase is a known downstream target of mGluR5 [26–28]. Our findings using cultured neurons show that the interaction between N-cadherin and β-catenin is modulated by mGluR5 in a Src kinase-dependent manner, since the inhibition of mGluR5 by CTEP or Src by A419259 increased the interaction between β-catenin and N-cadherin. Moreover, Src kinase is likely responsible for N-cadherin phosphorylation at Y860, as the kinetics of Src phosphorylation correspond with N-cadherin phosphorylation and its association with β-catenin. The change in DHPG-evoked N-cadherin phosphorylation required at least 60 mins of exposure to be detectable and therefore, it is likely that discerning changes in the N-cadherin and β-catenin association after DHPG exposure may require more than 60 mins. This may explain why DHPG did not reduce the co-immunoprecipitation of N-cadherin with β-catenin. It is noteworthy that DHPG is a Group I mGluR agonist and can potentially activate mGluR1 [51] . However, it is evident that we detect opposite changes in N-cadherin and Src phosphorylation as well as REST and SNAP-25 expression when cultures were exposed to CTEP indicating that DHPG-induced changes in neuronal cultures are mGluR5-mediated. More so, the effects of CTEP on REST/NRSF signaling were more robust compared to DHPG that can be possibly attributed to the constitutive activity of the receptor and the inverse agonistic properties of CTEP [52, 53].

We have also validated our neuronal culture findings in vivo and detected a substantial increase in mGluR5-mediated Src and N-cadherin phosphorylation in brain lysates from zQ175 mice that was sensitive to treatment with CTEP. Our findings are in line with previously published work in HN33 cells where the expression of polyglutamine-expanded huntingtin is associated with ∼5-fold increase of Src phosphorylation and induces the translocation of activated Src from cytoplasm to cell membrane [54]. Thus, it is possible that mHTT enhances mGluR5-dependent Src activation and triggers tyrosine-phosphorylation of its targets, such as N-cadherin. Because the antagonism of mGluR5 in zQ175 mice improves the motor phenotype and disease pathology and normalizes Src and N-cadherin phosphorylation, it is likely that aberrant Src/REST signaling is one of the mechanisms by which mGluR5 contribute to HD pathophysiology. It is worth noting that autophagy can play a role in the degradation process of REST/NRSF and hence, determines its nuclear availability [30, 31]. More so, mGluR5 is known to regulate autophagy [19, 55, 56], and indeed we detected an increase in REST/NRSF expression in DHPG-treated neurons and vehicle-treated zQ175 mice as well as a reduction in REST/NRSF expression in CTEP-treated neurons and CTEP-treated zQ175 mice. Thus, it is possible that regulation of autophagy may be another mechanism by which mGluR5 modulates the expression and nuclear availability of REST/NRSF in neurons.

**Fig. 8:**
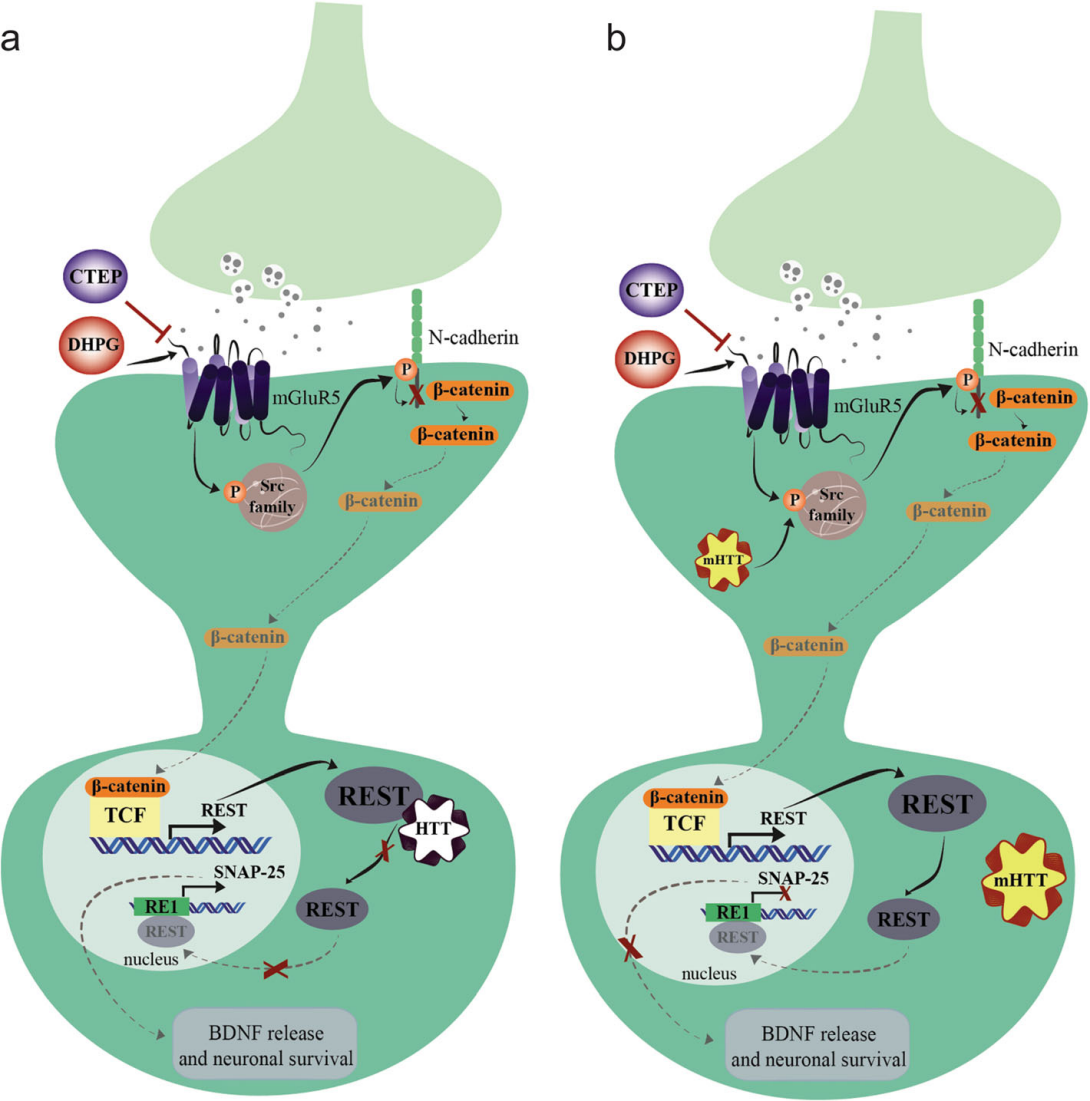
Schematic representation of the proposed model for the modulation of REST/NRSF signaling by mGluR5 and mHTT. **a** Shown is a schematic for mGluR5 signaling modulation by both mGluR5 agonist, DHPG, and the mGluR5-selective negative allosteric modulator, CTEP in the presence of HTT. mGluR5 induces Src family phosphorylation that phosphorylates N-cadherin at Y860, and this phosphorylation site in N-cadherin disrupts its interaction with β-catenin in cellular membrane. Then, β-catenin is released to cytoplasm and translocates to the nucleus, which becomes available to bind the TCF/LEF family of transcription factors to induce target gene expression, such as REST/NRSF. Under basal conditions, HTT sequesters REST/NRSF in the cytoplasm, thereby preventing it from forming the nuclear co-repressor complex at the RE1/NRSE nuclear site, allowing the transcription of REST/NRSF target gene, such as SNAP-25. SNAP-25 could affect the brain-derived neurotrophic factor (BDNF) release, supporting the survival of the striatal medium-sized spiny neurons. **b** The presence of mHTT enhances mGluR5-dependent Src activation, which culminates an increase of N-cadherin phosphorylation. Also, mHTT cannot retain REST/NRSF in the cytosol, causing the pathological entry of REST/NRSF into the nucleus, reducing SNAP-25 gene transcription and potentially BDNF release, which is correlated to neuronal death

In summary, although the contribution of the impaired Wnt canonical pathway to the pathology of HD has been reported previously [57–59], this study highlights a potential mechanistic link between mGluR5 and Wnt pathway and its contribution to HD pathology. We show that mGluR5 via Src kinase can regulate the assembly of the N-cadherin/ β-catenin complex and, as a consequence modulates the expression of REST/NRSF and of its downstream gene targets. Moreover, mHTT can alter Src activity and the expression of REST/NRSF and its target genes (Fig. 8), which can be reversed following either mGluR5 pharmacological blockade or genetic deletion. Thus, enhanced efforts should be directed towards exploiting the impact of mGluR5 on REST/NRSF-mediated gene expression, as this pathway may provide a conserved pathophysiological mechanism between HD and other neurodegenerative diseases.

## Materials and methods

### Reagents

CTEP was purchased from Axon Medchem and DHPG and A419259 from Tocris. Horseradish peroxidase (HRP)–conjugated anti-rabbit immunoglobulin G secondary antibody was from Bio-Rad. Neurobasal medium, N2 and B27 supplements, GlutaMAX (50 mg/ml penicillin and 50 mg/ml streptomycin), TRIzol, Nuclease-Free Water, and Power SYBR® Green PCR Master Mix were purchased from Thermo Fisher Scientific. Rabbit anti-REST (07–579) was from Merck. Rabbit anti-Src family (pY416; 2101) was from Cell Signaling Technology. Rabbit anti-N-cadherin (ab18203), phospho-N-cadherin (pY860; ab119752), β-catenin (ab6302), and SNAP25 (ab5666) were from Abcam. Reagents used for Western blotting were purchased from Bio-Rad, and all other biochemical reagents were from Sigma-Aldrich.

### zQ175 mice and drug administration

All animal experimental protocols were approved by the University of Ottawa Institutional Animal Care Committee and were in accordance with the Canadian Council of Animal Care guidelines. Animals were individually caged and housed under a constant 12-h light/dark cycle and given food and water ad libitum. Heterozygous zQ175 HD mice were obtained as a courtesy of CHDI Foundation from The Jackson Laboratory (stock #370476) and bred to establish littermate-controlled male wild-type (WT). zQ175 knockin mice carry ~ 188 CAG repeat expansions. Groups of 12 male wild-type and zQ175 mice were aged to 12 months of age, and 6 mice from each group were treated every 48 h with either vehicle [dimethyl sulfoxide (DMSO) in chocolate pudding] or CTEP (2 mg/kg; dissolved in DMSO and then mixed with chocolate pudding) for 12 weeks. This drug dose was calculated weekly on the basis of weight and is consistent with the dose given to fragile X and Alzheimer’s disease mice [60, 61]. At the end of the 12-week treatment, mice were sacrificed by exsanguination, and the brains were collected and randomized for western blot analysis.

### BACHD/mGluR5^−/−^ (double mutant)

FVB/NJ (wild-type, RRID: IMSR_JAX:001800) and FVB/N-Tg (HTT*97Q) IXwy/J (BACHD) transgenic mice [34] and mGlu5R knockout B6; 129-Grm5tm1Rod/J (mGluR5^−/−^) mice were purchased from The Jackson Laboratory (Bar Harbor, USA). For the generation of the double mutants, mGluR5^−/−^ mice and BACHD mice were crossed, obtaining the F1 parental lineage. Afterwards, F1 mice were crossed to obtain littermate male mice at the ages of 6 and 12 of WT, mGluR5^−/−^, BACHD and BACHD/mGluR5^−/−^ (double mutant). Mice were housed in an animal care facility at 23 °C on a 12 h light/12 h dark cycle with food and water provided ad libitum. All mice that euthanized in this study were first anesthetized with ketamine/xylazine (80/8 mg/kg) i.p. before cervical dislocation and the brains were collected, dissected and randomized for PCR and immunoblotting analyses. All animal experimental protocols were conducted in accordance with the Universidade Federal de Minas Gerais Ethics Committee on Animal Use, CEUA, 234/2016.

### Neuronal primary culture preparation

Neuronal cultures were prepared from the corticostriatal region of WT E15 embryo brains. After dissection, corticostriatal tissue of each embryo was digested by trypsin followed by cell dissociation using a fire-polished Pasteur pipette. Cells were plated on poly-L-ornithine coated dishes in Neurobasal medium supplemented with N2 and B27 supplements, 2 mM GlutaMAX, 50 μg/ml penicillin, and 50 μg/ml streptomycin (Thermo Fisher Scientific). Cells were incubated at 37 °C and 5% CO_2_ in a humidified incubator and cultured for 12 to 15 days with medium replenishment every 4 days at the day of the experiment. Cell were starved in Hank’s balanced salt solution (HBSS) for 1 h. Cells were then treated with CTEP or 100 μM DHPG for 10 min, 30 min, 60 min, 180 min and 360 min and 1 μM A419259 for 60 min at 37 °C, as indicated in the Figure legend. Following treatment, neuronal cultures were collected for immunoblotting and coimmunoprecipitation.

### Immunoblotting

Mouse brains were dissected and lysed in ice-cold triton lysis buffer [50 mM tris (pH 8.0), 150 mM NaCl, and 1% Triton X-100]. Neuronal primary cultures obtained from WT embryos were lysed in ice-cold RIPA buffer [0.15 M NaCl, 0.05 M tris-HCl, pH 7.2, 0.05 M EDTA, 1% Nonidet P40, 1% Triton X-100, 0.5% sodium deoxycholate, 0.1% SDS]. Both buffers contained protease inhibitors (1 mM AEBSF [4-(2 aminoethyl) benzenesulfonyl fluoride hydrochloride], leupeptin (10 μg/ml), and aprotinin (2.5 μg/ml)) and phosphatase inhibitors (10 mM NaF and 500 μM Na3VO4) and all samples were centrifuged at 15,000 rpm at 4 °C for 15 min. The supernatant was collected, and the total protein levels were quantified using Bradford protein assay (Thermo Fisher Scientific). Samples were prepared by adding 3x loading buffer containing β-mercaptoethanol to homogenates containing 30–70 μg of total proteins. Samples were then boiled for 10 min at 95 °C, resolved by electrophoresis on a 7.5% SDS–polyacrylamide gel and transferred onto nitrocellulose membranes (Bio-Rad). Membranes were blocked in tris-buffered saline (pH 7.6) containing 0.05% Tween 20 (TBST) and 5% nonfat dry milk for 2 h at room temperature and then incubated overnight at 4 °C with primary antibodies (1:1000) diluted in TBST containing 1% nonfat dry milk. Membranes were then incubated with secondary antibodies (anti- rabbit/mouse) diluted (1:5000) in TBST containing 1% nonfat dry milk for 1 h. Membranes were washed in TBST and bands were detected and quantified using a Bio-Rad chemiluminescence system.

### Quantitative RT-qPCR

RNA from cortical samples of mice at 6 and 12 months of age was isolated using TRIzol reagent as per manufacturer’s instructions (Thermo Scientific). RNA was resuspended in of nuclease-free water, and its concentration was analyzed by spectrophotometer (NanoDrop™, Thermo Scientific). cDNAs were prepared from 2 μg of total RNA extracted and RT-qPCR was performed from 10 × diluted cDNA using Power SYBR Green PCR Master Mix in the QuantStudio7 Flex real-time PCR system platform (Applied Biosystems). mRNA levels of REST/NRSF and SNAP25 were quantified using the following primers: REST (forward: 5′-CATGCTGATTAGAGGCCACA-3′; reverse: 5′GTGCGAACTCACACAGGAGA -3′); SNAP25 (forward: 5′ GCCTTCTCCATGATCCTGTC − 3′; reverse: 5′- CTTCATCCGCAGGGTAACAA-3′). Changes in gene expression were determined with the 2^−ΔΔCt^ method using actin as a housekeeping gene.

### Co-immunoprecipitation

Neuronal primary cultures obtained from WT embryos were lysed in ice cold triton lysis buffer [0.5 M HEPES, 2.5 M NaCl, 0.5 M MgCl2, 0.5 M EDTA, 0.2% Triton X-100, pH 7.4] containing protease inhibitors. Lysates were rotated for 1 h at 4 °C and centrifuged to pellet insoluble material. Precleared supernatant was incubated with anti-β-catenin antibody to immunoprecipitate N-cadherin. Following this incubation, freshly washed protein G-sepharose beads were added to lysate/antibody mixture and samples were rotated for 2 h at 4 C. Beads were washed three times with phosphate buffered saline, eluted with 3x SDS sample buffer containing β-mercaptoethanol and analyzed by immunoblotting.

### Statistical analysis

Means ± SEM are shown for the number of independent experiments indicated in figure legends. GraphPad Prism was used to analyze data for statistical significance. Statistical significance (*p* < 0.05) was determined by one-way or two-way analysis of variance (ANOVA) testing followed by Fisher’s LSD as indicated in each figure legend.

## Data Availability

All data generated or analyzed during this study are included in this published article.

## Abbreviations

BACHD: Bacterial artificial chromosome (BAC) Huntington’s disease
BDNF: Brain-derived neurotrophic factor
CTEP: 2-chloro-4-((2,5-dimethyl-1-(4-(trifluoromethoxy)phenyl)-1H-imidazol-4-yl)ethynyl) pyridine
DHPG: (S)-3,5-Dihydroxyphenylglycine
HD: Huntington’s disease
HTT: Huntingtin
mGluR5: Metabotropic glutamate receptor 5
mHTT: Mutant huntingtin
NAM: Negative allosteric modulator
REST/NRSF: Repressor element 1-silencing transcription factor/neuron-restrictive silencer factor
RE1/NRSE: Neuron-restrictive silencer element
SNAP-25: Synaptosomal nerve-associated protein-25
